# IL-15 promotes human myogenesis and mitigates the detrimental effects of TNFα on myotube development

**DOI:** 10.1038/s41598-017-13479-w

**Published:** 2017-10-11

**Authors:** Mary F. O’Leary, Graham R. Wallace, Andrew J. Bennett, Kostas Tsintzas, Simon W. Jones

**Affiliations:** 1 Institute of Inflammation and Ageing, MRC-ARUK Centre for Musculoskeletal Ageing Research, University of Birmingham, Birmingham, UK; 2 FRAME Alternatives Laboratory, Faculty of Medicine & Health Sciences, University of Nottingham, Nottingham, UK; 3 MRC-ARUK Centre for Musculoskeletal Ageing Research, Faculty of Medicine & Health Sciences, University of Nottingham, Nottingham, UK

## Abstract

Studies in murine cell lines and in mouse models suggest that IL-15 promotes myogenesis and may protect against the inflammation-mediated skeletal muscle atrophy which occurs in sarcopenia and cachexia. The effects of IL-15 on human skeletal muscle growth and development remain largely uncharacterised. Myogenic cultures were isolated from the skeletal muscle of young and elderly subjects. Myoblasts were differentiated for 8 d, with or without the addition of recombinant cytokines (rIL-15, rTNFα) and an IL-15 receptor neutralising antibody. Although myotubes were 19% thinner in cultures derived from elderly subjects, rIL-15 increased the thickness of myotubes (MTT) from both age groups to a similar extent. Neutralisation of the high-affinity IL-15 receptor binding subunit, IL-15rα in elderly myotubes confirmed that autocrine concentrations of IL-15 also support myogenesis. Co-incubation of differentiating myoblasts with rIL-15 and rTNFα, limited the reduction in MTT and nuclear fusion index (NFI) associated with rTNFα stimulation alone. IL-15rα neutralisation and rTNFα decreased MTT and NFI further. This, coupled with our observation that myotubes secrete IL-15 in response to TNFα stimulation supports the notion that IL-15 serves to mitigate inflammatory skeletal muscle loss. IL-15 may be an effective therapeutic target for the attenuation of inflammation-mediated skeletal muscle atrophy.

## Introduction

Loss of skeletal muscle mass with age (sarcopenia), injury and illness is a major contributor to frailty and disability in the elderly^1,2^. Importantly, recent studies in the mouse suggest that IL-15, a 14 kDa four-helix bundle cytokine may play a central role in the growth and maintenance of skeletal muscle^3–6^. However, to date, no studies have examined the expression or functional role of IL-15 in human derived skeletal muscle tissue or primary cells.

Stimulation of murine C2C12 myoblasts with recombinant IL-15 (rIL-15) increases myoblast proliferation and myosin heavy chain expression, promoting the development of larger myotubes^7^. Furthermore, *ex-vivo* stimulation of rat extensor digitorum longus muscle with rIL-15 decreased skeletal muscle proteolytic rate^8^, suggesting an anti-atrophic effect of IL-15 on muscle tissue. Indeed, it is suggested that IL-15 may play an important key role in the maintenance of muscle mass in the presence of atrophic stimuli. For example, rIL-15 ameliorated the induction of protease (cathepsin L) activity in TNFα and dexamethasone stimulated C2C12 myotubes^9^. Furthermore, in an experimentally-induced sepsis mouse model, rIL-15 reduced the mRNA expression of the E3 ligases MAFbx and MuRF-1 which ubiquitinate and target proteins for proteasomal degradation^9^. rIL-I5 treatment of Yoshida AH-130 ascites hepatoma rats decreased skeletal muscle protein degradation 8-fold and significantly limited loss of body mass as well as soleus and tibalis muscle mass^10^. It has been proposed that IL-15 is a compensatory factor, expressed by skeletal muscle in order to mitigate conditions promoting skeletal muscle atrophy^11^.

Importantly, current evidence from *in vivo* human studies points to a crucial role for myogenesis in adult skeletal muscle maintenance, hypertrophy and remodelling in response to disuse atrophy as well as injurious and non-injurious exercise^12^. Therefore, in this study we used a model of adult human myoblast differentiation into myotubes to determine the effect of IL-15 on myogenesis. We further sought to establish whether the purported myogenic effects of IL-15 are conserved in elderly human skeletal muscle, since IL-15 signalling may represent an important pathway for the identification and development of therapeutic approaches designed to preserve the loss of skeletal muscle mass and quality in ageing. Given the propensity of the elderly to develop sarcopenia, we hypothesised that myogenic cultures derived from the skeletal muscle of elderly individuals would be more resistant to the hypertrophic effects of IL-15 than those of young individuals. Finally, we sought to examine whether IL-15 could protect primary human myotube development from the deleterious effects of TNFα, a pro-inflammatory cytokine implicated in the pathogenesis of sarcopenia^13^ and cachexia in chronic illness^14^.

## Results

### Effect of IL-15 on human myotube development and myogenic gene expression

To examine the effect of IL-15 on human myotube development, myoblasts from young subjects were differentiated for 8 d in the presence of recombinant human IL-15 (rIL-15) at 1, 25 and 100 ng/mL. The resulting myotubes were fixed, immunofluorescence (IF) stained for desmin and counterstained with DAPI. Plates were imaged and myogenesis quantified by determining myotube thickness (MTT) and nuclear fusion index (NFI).

**Figure 1:**
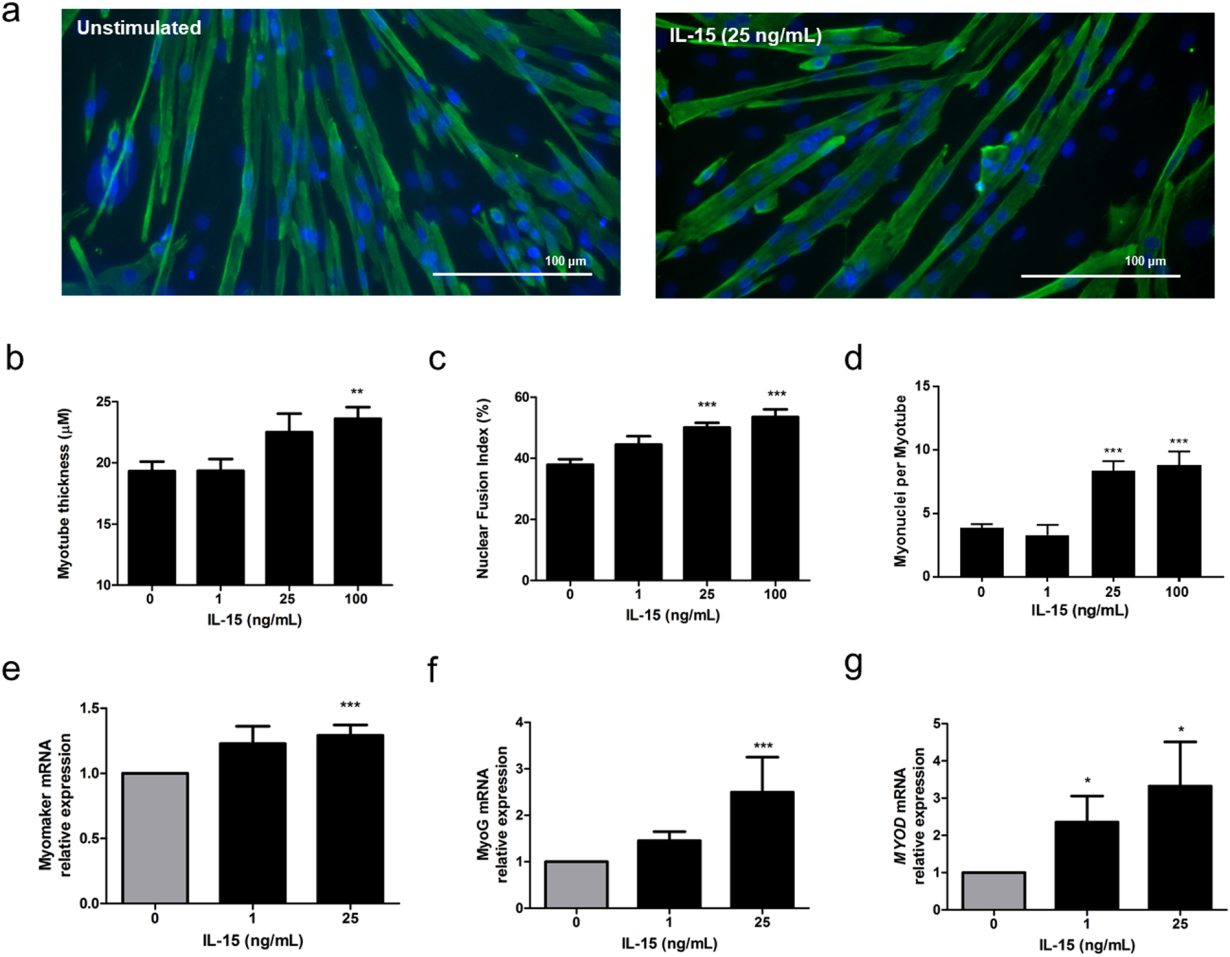
Recombinant IL-15 stimulation of differentiating primary human myoblasts enhances myotube formation and promotes myogenic gene expression. Subconfluent myoblasts were switched to differentiation medium containing the indicated concentrations of IL-15 for 8 d. Media were renewed every 2 d. Myotubes were fixed, immunofluorescence stained for desmin and with DAPI and imaged on an epifluorescence microscope. (**a**) Representative images at 20x magnification. (**b**) Myotube thickness data represents the mean ± SEM of 450 total measurements taken at 63x magnification from 90 myotubes per treatment condition (n = 3 biological replicates). **p < 0.01 vs. unstimulated (0 ng/mL) condition by Mann-Whitney U test with post-hoc Holm’s sequential Bonferroni adjustment. (**c**) Nuclear fusion index data is expressed as mean ± SEM values from 45 images taken at 20x magnification from 3 biological replicates. ***p < 0.001 vs. unstimulated (0 ng/mL) condition by one-way ANOVA with post-hoc Bonferroni correction. (**d**) Number of myonuclei per myotube is expressed as mean ± SEM values from 15 images taken at 20x magnification from 3 biological replicates. (**e**) Primary human myoblasts were stimulated with recombinant IL-15 at the indicated concentrations for 8 days. Media were renewed every 2 d. ***p < 0.001 vs.unstimulated condition by one-way ANOVA with post-hoc Bonferroni correction. (**f**,**g**) Subconfluent myoblasts were switched to differentiation medium containing the indicated concentrations of IL-15 for 8 d. Media were renewed every 2 d *p < 0.05, ***p < 0.001 vs. unstimulated condition by Mann-Whitney U test with post-hoc Holm’s sequential Bonferroni adjustment. Data expressed as mean ± SEM. 3 technical replicates (each assayed in triplicate) per biological replicate, 3 biological replicates

rIL-15 (100 ng/mL) significantly increased the MTT of differentiated myotubes by 22 ± 5% (p < 0.01) (Fig. 1a,b). Stimulation of differentiating myotubes for 8 d with either 25 ng/mL or 100 ng/mL rIL-15 also enhanced the NFI (35 ± 4%, p < 0.0001; 45 ± 7%, p < 0.0001 at 25 and 100 ng/mL respectively), compared to unstimulated controls (Fig. 1c). Furthermore, the average number of myonuclei in each myotube was enhanced by rIL-15 stimulation (114 ± 20%, p < 0.0001; 128 ± 27%, p < 0.0001 at 25 and 100 ng/mL respectively) (Fig. 1d).

We then examined whether IL-15 affected the mRNA expression of known mediators of myogenesis (namely myomaker, and the myogenic transcription factors MyoD, myogenin and Myf5) in un-differentiated myoblasts and in myoblasts during their differentiation into myotubes. Firstly, myoblasts were stimulated with rIL-15 (at 1ng/mL or 25 ng/mL) or left unstimulated for 8 d, at which time they had reached 90% confluence. Media were renewed every 2 d. Stimulation of myoblasts with rIL-15 (25 ng/mL) induced a small (1.3-fold) but highly significant (p < 0.0001) increase in the expression of myomaker, a cell membrane protein that is essential for myoblast fusion (Fig. 1e). Notably, this alteration in myomaker expression was not accompanied by changes in the expression of either *MYF5, MYOG* or *MYOD1* expression (Supplementary Fig. 1).

In a separate experiment, myoblasts at 90% confluence were switched to differentiation medium, or to differentiation media containing rIL-15 (at 1 ng/mL or 25 ng/mL). After 8 d of differentiation, myogenic regulatory factor gene expression was quantified by RT-qPCR. rIL-15 increased the gene expression of *MYOG* (1.45-fold, p = 0.09; 2.5-fold, p < 0.0001 at 1 and 25 ng/mL respectively) *and MYOD* (2.36 fold, p = 0.02; 3.32-fold, p < 0.05 at 1 and 25 ng/mL respectively) in differentiated myotubes (Fig. 1f,g).

### Recombinant IL-15 Stimulation of Differentiating Primary Human Myoblasts Partially Reverses TNFα-induced Inhibition of Myogenic Differentiation in Young and Old Myotubes

Next we sought to determine whether myogenic cultures derived from elderly subjects were also sensitive to the pro-myogenic effects of rIL-15 and whether rIL-15 was capable of reversing the deleterious effects of recombinant TNFα (rTNFα) on myogenesis using an rTNFα concentration representative of those found systemically in cachexia^15,16^.

**Figure 2:**
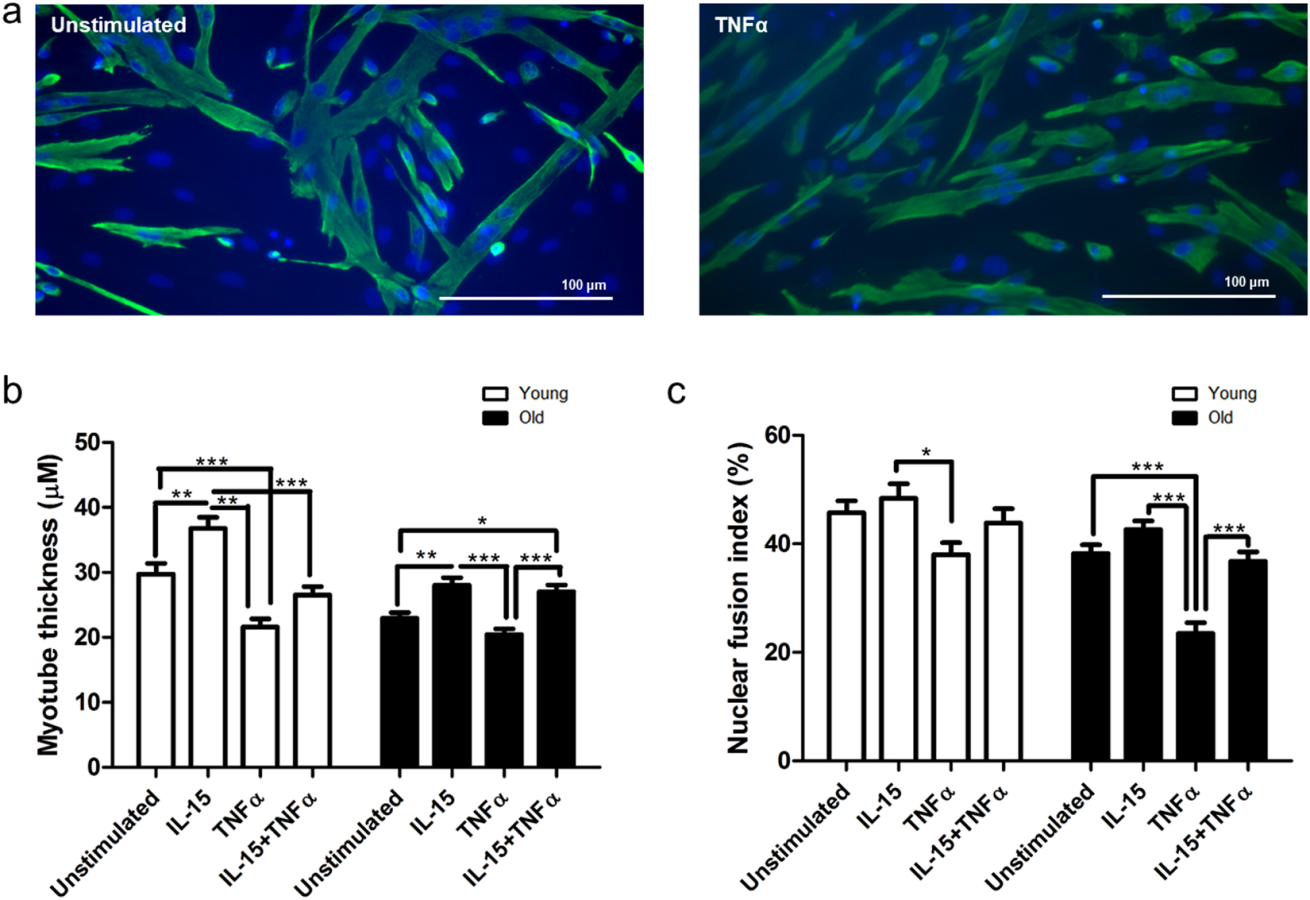
Recombinant IL-15 stimulation of differentiating primary human myoblasts partially reverses TNFα-induced inhibition of myogenic differentiation of cells from both young and old individuals. Subconfluent myoblasts were switched to differentiation medium containing the indicated recombinant cytokines (IL-15, 25 ng/mL; TNFα, 1 ng/mL) for 8 d. Media were renewed every 2 d. Myotubes were fixed, immunofluorescence stained for desmin and DAPI and imaged on an epifluorescence microscope. (**a**) Representative images at 20x magnification. (**b**) Myotube thickness data represents the mean ± SEM of 450 total measurements taken at 63x magnification from 90 myotubes per treatment condition (from 3 biological replicates). (**c**) Nuclear fusion index data represents the mean ± SEM values from 45 images taken at 20x magnification (from 3 biological replicates). *p < 0.05, **p < 0.01, ***p < 0.001 vs. unstimulated condition by 2-way ANOVA with post-hoc Bonferroni correction.

Differentiating myoblasts from young and old subjects were stimulated with either rTNFα (1 ng/mL) alone, rIL-15 (25 ng/ml) ± rTNFα (1 ng/mL) or were left unstimulated (controls) over 8 d. Media were renewed every 2 d. The resulting myotubes were fixed, IF stained and imaged as before (Fig. 2a). Myotubes were 19% thinner in cultures derived from elderly subjects. However, rIL-15 increased the MTT of myotubes from elderly donors (22 ± 5%, p = 0.02), indicating that they are sensitive to the hypertrophic effects of IL-15 that we observed in the young (Fig. 2b). In young myotubes, rTNFα alone induced a 30 ± 5% decrease in myotube thickness (p < 0.0001) compared to unstimulated control myotubes. However, co-incubation of differentiating myoblasts with rIL-15 and rTNFα partially reversed this effect, limiting loss of MTT to 11 ± 6% of the control, a significant improvement compared to the rTNFα alone condition (p = 0.04). In old myotubes, the decrease in MTT induced by TNFα was not significant, however, myotubes co-stimulated with TNFα and rIL-15 condition were significantly thicker (29 ± 7%, P = 0.001) than their TNFα-stimulated counterparts (Fig. 2b).

rIL-15 alone did not significantly increase the NFI in old myotubes or in this cohort of young myotubes. However, in both age groups co-incubation of differentiating myoblasts with rIL-15 and rTNFα completely reversed TNFα-induced reductions in NFI, resulting in NFI values which did not differ significantly from the control (Fig. 2c). rIL-15 stimulation of proliferating myoblasts had no effect on their proliferation or survival rates (Supplementary Fig. 2). Furthermore, in differentiated cultures the number of nuclei per field of view did not differ significantly between stimulation conditions, suggesting that their effects on MTT and NFI were not due to differences in cell survival, proliferation or errors in cell seeding density (Supplementary Fig. 2). As before (Fig. 1), 25 ng/mL rIL-15 induced a substantial and significant increase in the average number of myonuclei in each myotube. However, myotubes formed in the presence of rTNFα, did not differ from unstimulated controls in their myonuclear number, irrespective of whether they were co-stimulated with rIL-15 (Supplementary Fig. 2).

**Figure 3:**
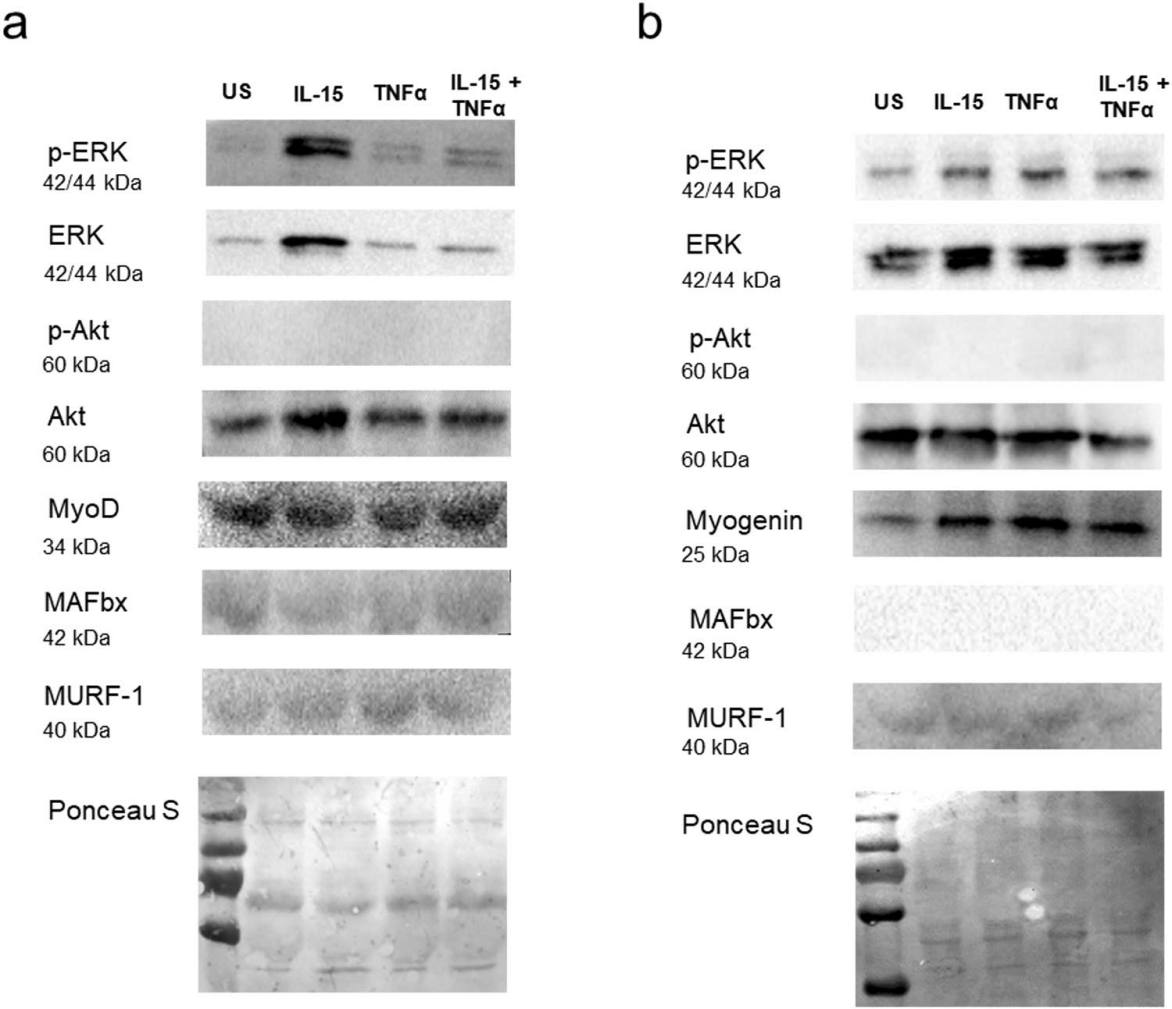
Recombinant IL-15 stimulation of primary human myogenic cultures induces molecular changes that suggest it has pro-myogenic actions. (**a**) Commercially available subconfluent myoblasts from a female aged 21 yr were switched to growth medium containing the indicated recombinant cytokines (IL-15, 25 ng/mL; TNFα, 1 ng/mL) for 2 d. The expression of the indicated proteins was determined by immunoblotting. Full-length blots are presented in Supplementary Figure 6. Blots are representative of duplicate independent experiments. (**b**) Commercially available subconfluent myoblasts from a female aged 21 yr were switched to differentiation medium containing the indicated recombinant cytokines (IL-15, 25 ng/mL; TNFα, 1 ng/mL) for 2 d. The expression of the indicated proteins was determined by immunoblotting. Full-length blots are presented in Supplementary Figure 6. Blots are representative of duplicate independent experiments.

Having observed pro-myogenic effects of rIL-15 both in the presence and the absence of a pro-inflammatory (rTNFα) stimulus, we sought further molecular corroboration for our observations in both undifferentiated myoblasts and in differentiating cultures. Subconfluent myoblasts were switched to growth medium containing the recombinant cytokines (IL-15, 25 ng/mL; TNFα, 1 ng/mL) or were left unstimulated for 2 d. Immunoblotting demonstrated that rIL-15 induced the expression of total ERK 1/2 (p44/42 MAPK), phosphorylated-ERK (Thr202/Tyr204) and total Akt. A modest induction of total ERK and phosphorylated ERK expression was evident in cultures that were co-stimulated with rIL-15 and TNFα (Fig. 3a). No changes in MyoD, MAFbx or MURF-1 expression were observed and myogenin was not detected in these undifferentiated cultures (Fig. 3a).

Secondly, subconfluent myoblasts were switched to differentiation medium containing the recombinant cytokines (IL-15, 25 ng/mL; TNFα, 1 ng/mL) or were left unstimulated for 2d, at which time myotubes begin to form in our cultures. Similar to the undifferentiated cultures, stimulation with rIL-15 induced an increase in total ERK and phosphorylated ERK expression (Fig. 3b). As expected, myogenin protein could be detected in these differentiated cultures, and its expression was increased in cultures stimulated with either rIL-15 or rTNFα, compared to unstimulated myotubes (Fig. 3b). In addition, rIL-15 induced an increase in MHC protein content (Supplementary Fig. 4A), despite not significantly increasing total muscle protein synthesis (Supplementary Fig. 4B), as measured using the non-radioactive surface sensing of translation (SUnSET) method^17,18^.

### Elderly individuals display increased skeletal muscle IL-15 expression and increased plasma IL-15 concentrations

**Figure 4:**
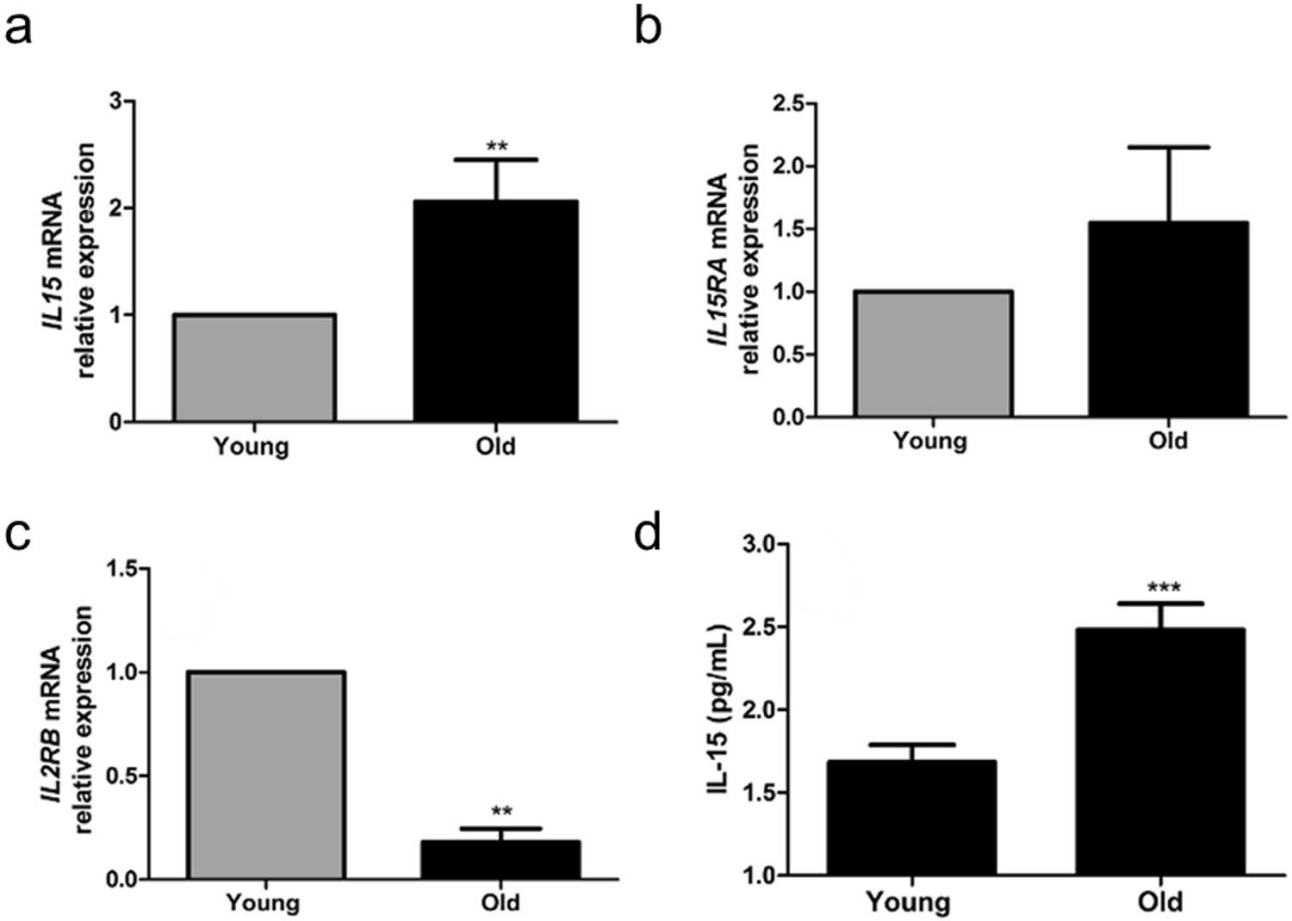
Elderly individuals display increased skeletal muscle IL-15 expression and increased plasma IL-15 concentrations. (**a**,**b**,**c**) *IL15*, *IL15RA* and *IL2RB* gene expression were quantified in skeletal muscle tissue by RT-PCR. **p < 0.01, vs. unstimulated condition by Mann-Whitney U test, N = 5. (**d**) Plasma IL-15 concentrations were assayed by ELISA. ***p < 0.001 vs. the unstimulated condition by t-test. Data expressed as mean ± SEM, N = 10.

Cognisant of the suggestion that IL-15 expression increases in order to compensate for conditions that promote skeletal muscle loss^11^, we quantified circulatory levels of IL-15, as well as the expression of IL-15 and its receptors in young and old skeletal muscle tissue derived from skeletal muscle biopsies. Skeletal muscle expression of *IL15* was 2-fold higher (Fig. 4a) in old subjects, compared to young subjects. Furthermore, circulatory concentrations of IL-15 were significantly greater in older subjects, being ~ 50% higher (p = 0.0006) in the old compared to the young (Fig. 4d). However, we also found that the IL-15 receptor signalling subunit *IL2RB* was decreased by ~80% in old skeletal muscle, compared to young, with no change in the expression of *IL15RA* (Fig. 4b,c).

### TNFα induces IL-15 expression in and its secretion from primary human myotubes

Given that IL-15 reverses the anti-myogenic actions TNFα and that IL-15 has been proposed as compensatory agent that is expressed in response to atrophic stimuli^19^, we sought to establish whether an inflammatory stimulus could induce IL-15 secretion and expression in primary human myotubes.

**Figure 5:**
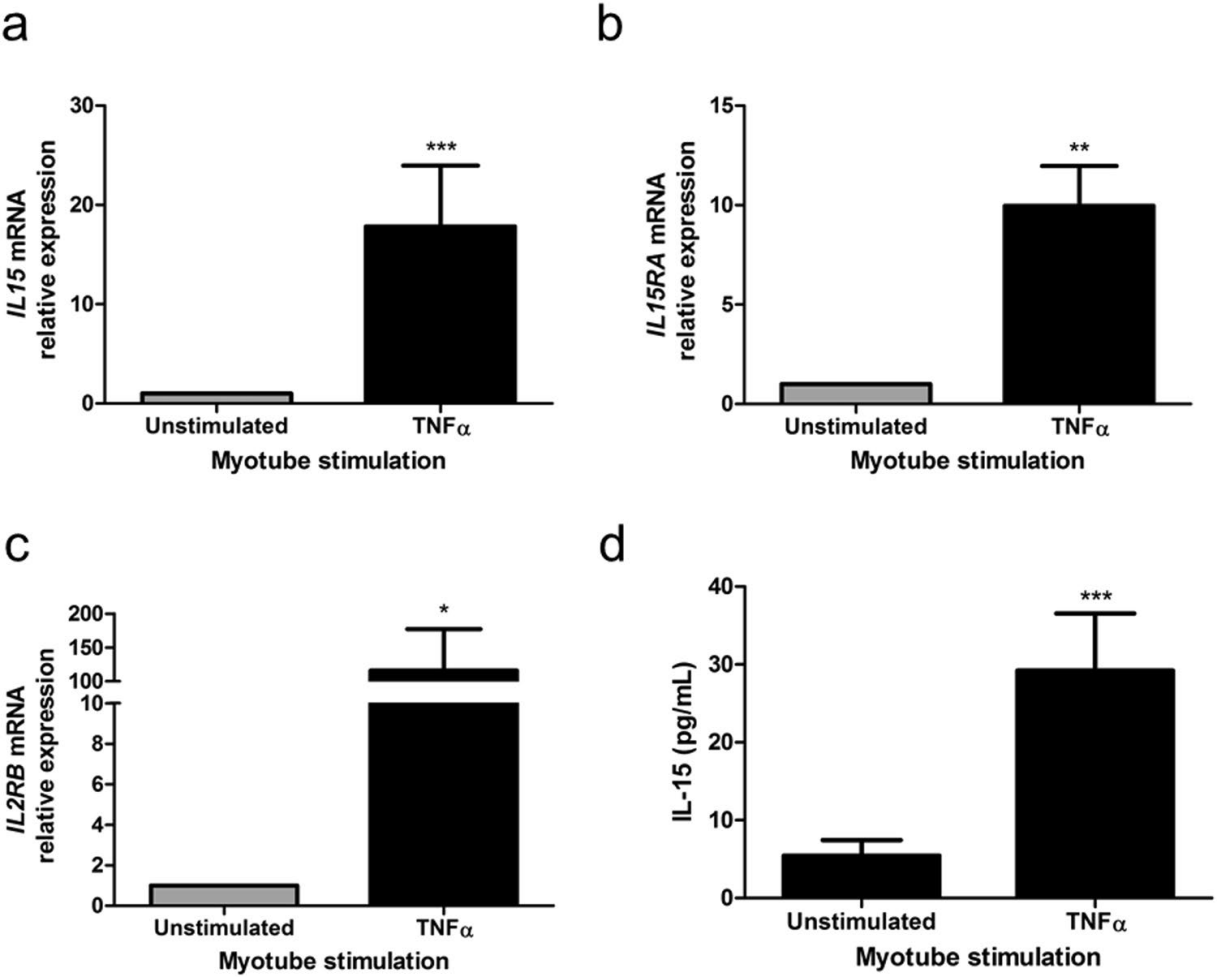
Stimulation of primary human myotubes with recombinant TNFα induces IL-15/L-15 receptor gene expression and the secretion of IL-15. Primary human myotubes were stimulated with recombinant TNFα (20 ng/mL) for 24 hours. (**a**,**b**,**c**) *IL15*, *IL15RA* and *IL2RB* gene expression were quantified by RT-PCR. **p < 0.01, ***p < 0.001 vs. unstimulated condition by Mann-Whitney U test. 3 technical replicates (each assayed in triplicate) per biological replicate, 3 biological replicates. (**d**) Supernatant IL-15 concentrations were assayed by ELISA. ***p < 0.001 vs. unstimulated condition by one-way ANOVA with post-hoc Bonferroni correction. Data expressed as mean ± SEM; 5 technical replications per biological replicate, 3 biological replicates.

Stimulation of primary human myotubes from young subjects with 20 ng/mL rTNFα for 24 h induced a 19-fold increase in myotube *IL15* expression (p = 0.0004) (Fig. 5a). Myotubes were found to express both *IL15RA* and *IL2RB*, and 24 h rTNFα stimulation significantly induced the expression of both *IL15RA* (10-fold; p = 0.0014), and *IL2RB* (126-fold; p = 0.01), relative to non-stimulated control myotubes (Fig. 5b,c). We also observed a corresponding 4-fold (p = 0.0006) increase in culture supernatant IL-15 concentration (Fig. 5d), indicating a significant increase in IL-15 secretion from rTNFα stimulated human myotubes.

### Antibody neutralisation of IL-15rα eliminates the myogenic effects of IL-15 and enhances the detrimental effects of TNFα on myotube development

Given our findings that IL-15 acts to facilitate myotube development in an inflammatory environment and that TNFα induces its secretion from primary human myotubes, we investigated whether disruption of IL15 binding to its receptor would cause a further deterioration in myogenesis in the presence of TNFα.

**Figure 6:**
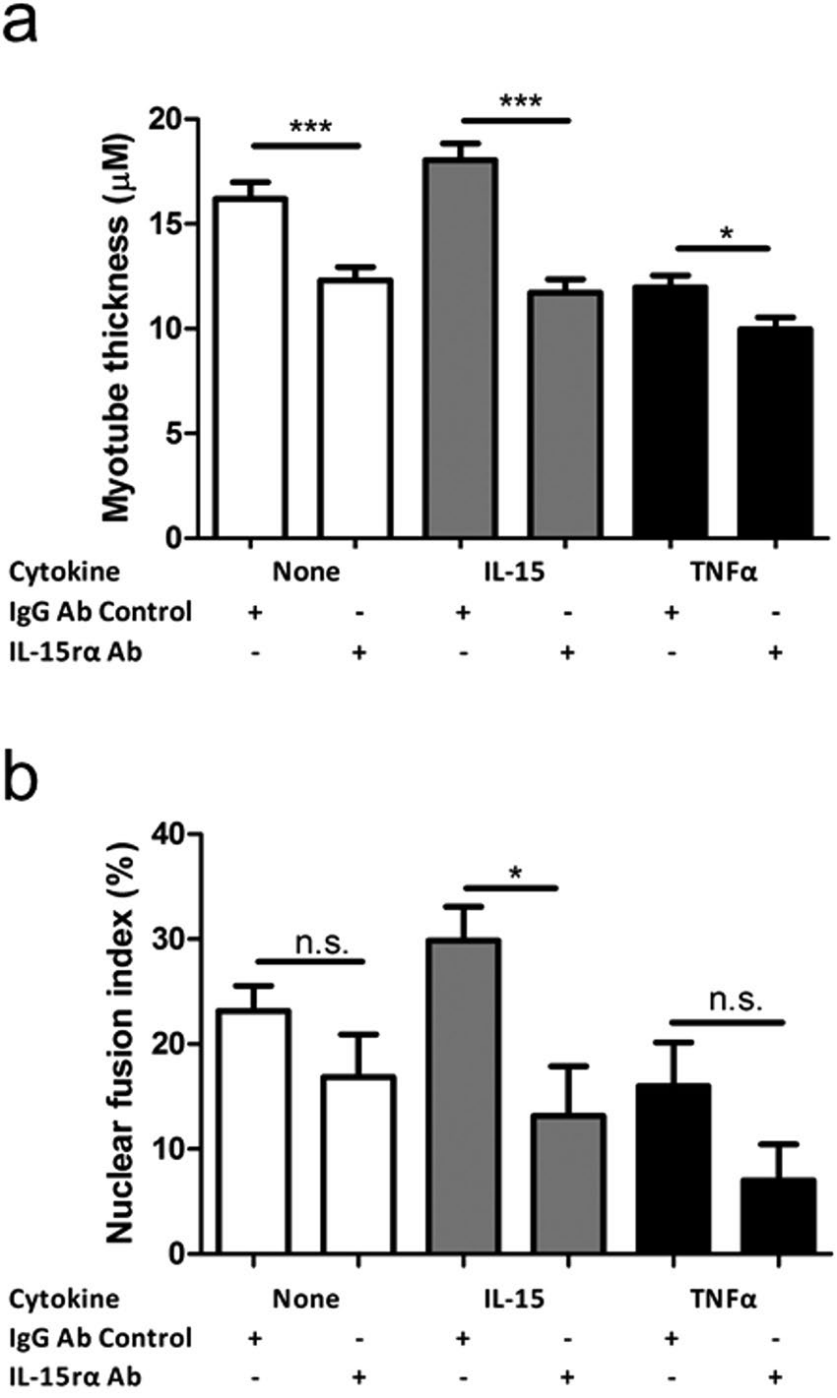
Antibody neutralisation of IL-15rα eliminates the myogenic effects of IL-15 and enhances the detrimental effects of TNFα on myotube development. Subconfluent myoblasts were switched to differentiation medium containing the indicated recombinant cytokines and antibodies (IL-15, 25 ng/mL; TNFα, 1 ng/mL; IL-15rα antibody, 1 µg/mL; IgG1 antibody, 1 µg/mL) for 8 d. Media were renewed every 2 d. Myotubes were fixed, immunofluorescence stained for desmin and DAPI and imaged on an epifluorescence microscope. (**a**) Myotube thickness data represents the mean ± SEM of 450 total measurements taken at 63x magnification from 90 myotubes per treatment condition (from 3 biological replicates). (**b**) Nuclear fusion index data represents the mean ± SEM values from 45 images taken at 20x magnification (from 3 biological replicates). *p < 0.05, ***p < 0.001 vs. IgG control condition by t-test.

We employed an antibody against the IL-15 receptor binding subunit, IL-15rα. Myoblasts from elderly subjects were differentiated for 8 d in the presence of IL-15 (25 ng/mL) or TNFα (1 ng/mL) with the further addition of an IL-15rα neutralising antibody (1ug/mL) or an IgG isotype control (1 ug/mL). Media were renewed every 2 d. The presence of the IL-15rα neutralising antibody significantly reduced the MTT of otherwise untreated (24 ± 4%, p = 0.003), IL-15 stimulated (35 ± 3%, p = <0.0001) and TNFα stimulated (17 ± 2%, p = 0.014) myotubes compared to their IgG isotype treated controls (Fig. 6a). The IL-15rα neutralising antibody had a similar effect on NFI, although the numerically considerable reduction in NFI observed in TNFα stimulated myotubes (56 ± 21%, p = 0.16) treated with a IL-15rα neutralising antibody did not reach significance (Fig. 6b). The average number of myonuclei in each myotube was not significantly altered by any treatment condition (Supplementary Fig. 5).

## Discussion

We have demonstrated for the first time that IL-15 enhances human myotube development and can partially protect against the deleterious effects of TNFα on myogenesis. Secretion of IL-15 by primary human myotubes derived from patients with polymyositis and dermatomyositis has been previously described^20^. Here we show for the first time that myotubes derived from the skeletal muscle of healthy subjects also secrete IL-15. These data are a significant advancement on previous *in vitro* work describing the effects of IL-15 on skeletal muscle growth and maintenance as we have used primary human myogenic cultures, rather than an immortalised murine cell line.

Our studies were primarily designed to firstly examine the functional effect of IL-15 in human myogenesis and revealed that IL15 had a marked effect on increasing MTT, NFI and the number of myonuclei per myotube (Fig. 1). The magnitude of these changes (a 22% increase in MTT at the 100 ng/mL dose of rIL-15) is considerable when placed in an integrated physiological context. A previous study in humans has shown that even a small reduction in quadriceps lean mass following limb immobilisation (5% over 2 weeks) is accompanied by a significant decline in isometric strength^21^. Furthermore, 12 weeks of progressive resistance training in older men increases MHC I fibre diameter by 20%, a change which is associated with a 55% increase in maximal isometric force production by individual muscle fibres^22^. We also observed that myotubes were 19% thinner in our cultures derived from elderly subjects compared to young subjects.

The IL-15-mediated increase in MTT is unlikely to be due to induction of myoblast hyperplasia as we found no effect of IL-15 on either the proliferation or viability of subconfluent myoblasts. This is in agreement with previous observations that IL-15 has no effect on proliferation, as measured by [^3^H]thymidine incorporation in C2C12 myoblasts^7^. Instead, the hypertrophic mechanism appears to be via an enhancement of myoblast fusion and the myogenic program, since we observed a >30% increase in the NFI and a greater than 2-fold increase in the number of myonuclei in myotubes formed under IL-15 stimulation conditions. This notion of enhanced myogeneis is supported by our finding of total Akt induction in IL-15-stimulated confluent myoblasts that were ready for myogenic differentiation. Induction of total Akt expression (in particular that of Akt2) is essential for full myotube development^23^. Furthermore, we observed an increase in total ERK 1/2 and phosphorylated-ERK expression in IL-15 stimulated myoblasts. Myogenic cultures appear to have a biphasic requirement for ERK1/2 activity; ERK 1/2 appears to inhibit differentiation in proliferating cultures, but its activity facilitates myotube formation in differentiating cultures^24^. Given that these myoblasts had reached a confluence sufficient for myogenic differentiation, it is reasonable to hypothesise that ERK 1/2 activation by IL-15 might promote their fusion. This requires further study.

Furthermore, IL-15 induced an increase in myomaker gene expression in confluent myoblasts that were ready for myogenic differentiation. Myomaker is a recently discovered muscle-specific cell membrane protein that has been reported to be essential for myoblast fusion and muscle regeneration^25,26^. Most recently, myomaker dependent myoblast fusion to existing myofibres has been shown to be essential for overload-induced hypertrophy to occur in mice^27^. The finding that myomaker expression increased in IL-15 stimulated myoblasts represents a potentially novel mechanism of myogenic action for IL-15 in human skeletal muscle. In chicken myoblasts, the myogenic regulatory factors (MRFs) MYOG and MYOD have been shown to bind to an E-box near the myomaker transcription start site, thus inducing myomaker expression^28^. Indeed, IL-15 induced an increase in the expression of both *MYOD* and *MYOG* in differentiated myotubes.

Ageing is associated with a loss of skeletal muscle mass and quality – sarcopenia^29^. Given that our myotubes from elderly subjects formed less well than those from their young counterparts, we hypothesised that elderly cultures might be less sensitive to the myogenic actions of IL-15. However, we observed no difference in the functional responsiveness to exogenous rIL-15 stimulation between old and young myotubes, demonstrating that IL-15 signalling is not functionally impaired in the elderly. Despite observing no difference in the functional responsiveness to exogenous rIL-15 stimulation between young and old myotubes we did observe that the skeletal muscle tissue expression of the signalling subunit of the IL-15 receptor (*IL2RB)* was found to be significantly reduced in the elderly, compared to the young subjects. However, the expression of the IL-15 receptor binding subunit, *IL15RA* was maintained. Given its high affinity for IL-15 compared to the signalling subunit. maintenance of *IL15RA* expression may compensate for the reduction in *IL2RB* expression.

Importantly, evidence from *in vivo* human studies suggest an important role for myogenesis in adult skeletal muscle^12^. Therefore, our finding that IL-15 also promotes myoblast fusion in developing primary human myofibres derived from old subjects is particularly pertinent. Furthermore, one purpose of neutralising the high-affinity IL-15 receptor binding subunit, IL-15rα in elderly myotubes was to confirm that the pro-myogenic activity of IL-15 in elderly individuals is not limited to the pharmacological doses of the recombinant cytokine used by ourselves and others^9,30^. Our neutralisation of the receptor was successful; it completely inhibited the myogenic actions of exogenous rIL-15 (Fig. 6). In otherwise unstimulated elderly myotube cultures, IL-15rα neutralisation had a deleterious effect on myogenesis, suggesting that autocrine IL-15 secretion in elderly myotubes is crucial to support optimal myotube formation.

Given the emerging importance of inflammation in the aetiology of sarcopenia^13^, and its pathological role in cachexia^14^ it is remarkable and of potential clinical importance that IL-15 proved capable of ameliorating the deleterious effects of TNFα on primary human myotube development, regardless of culture donor age (Fig. 2). TNFα is known to induce myotube atrophy^31^ and its plasma levels increase with age, independent of BMI^32^. Given the positive myogenic actions of IL-15, we hypothesise that the observed TNFα-mediated induction of IL-15 secretion and expression of *IL15* and its receptor subunits *IL15RA* and *IL2RB* (Fig. 4), may represent a homeostatic mechanism, whereby IL-15 is induced to facilitate skeletal muscle maintenance in the presence of inflammation e.g. in inflammaging or in chronic clinical conditions that are associated with cachexia^33^. Indeed, we observed that MTT was decreased to an even greater degree by TNFα in the presence of a neutralising IL-15rα antibody (Fig. 6a), supporting the notion that IL-15 serves as compensatory factor in highly inflammatory environments. The induction of IL-15 secretion from TNFα stimulated myofibres might also, in part, explain why concentrations of TNFα in the normal physiological range have been reported to support myotube formation^34^. It is important to note that despite clearly demonstrating the pro-myogenic actions of IL-15, we have not fully established the molecular mechanism by which IL-15 ameliorates the detrimental effects of TNFα on myogenesis. It may be that that IL-15 remains pro-myogenic in the presence of TNFα. However, our molecular signalling analysis showed that the IL-15-mediated induction of pERK and of total AKT was markedly diminished by the presence of TNFα (Fig. 3a,b). Furthermore, unlike with IL-15 stimulation alone, co-stimulation of differentiating myotubes with IL-15 and TNFα did not increase the number of myonuclei per myotube (Supplementary Fig. 2D,E).

In summary, we have provided evidence for the first time that IL-15 enhances *in vitro* human myogenesis and can partially protect against the deleterious effects of TNFα on human myotube development. Targeting the IL-15 signalling pathway may therefore be an effective therapeutic approach in combating skeletal muscle atrophy, particularly in inflammatory disease states.

## Experimental Procedures

### Subject Recruitment and Ethical Approval

Ten young healthy subjects (5 males and 5 females; age 21.8 ± 0.5; weight 59.6 ± 3.3 kg; BMI 21.4 ± 0.9 kg/m^2^) and five elderly healthy individuals (3 males and 2 females; age 69.9 ± 2.3; weight 69.7 ± 10.5 kg; BMI 23.8 ± 2.3 kg/m^2^) were enrolled in this study and gave written informed consent. The study was approved by the University of Nottingham Medical School Ethics Committee and was conducted in accordance with the guidelines of the Declaration of Helsinki. Samples for the quantification of plasma IL-15 were obtained from healthy young and old subjects in a study that was approved by the Birmingham East North and Solihull Research Ethics Committee (09/H1206/48) and all subjects gave their written informed consent before taking part in the study.

### Tissue Collection and Cell Culture

A vastus lateralis muscle biopsy was obtained from each subject using the suction-modified Bergström technique^35^ and the satellite cell population extracted. Briefly, samples were minced with a scalpel and digested on an orbital rotator for 15 min at 37 °C. 5 mL growth medium (Ham’s F10 with 2mM L-glutamine, 100 μg/mL penicillin/streptomycin, 20% FBS) was added and the digest centrifuged at 460 g for 5 min. The resulting pellets were resuspended in growth medium and incubated for 20 min at 37 °C, 5% CO_2_ in an uncoated T75 tissue culture flask. The cell suspension was removed to flasks or plates coated with 0.2% gelatin and the myoblasts cultured in growth medium. Myoblasts were subcultured (1:3) at 70% confluence by trypsin-induced dissociation from their vessel and were used experimentally at passages 2-4. Myoblasts at 90% confluence were differentiated to myotubes by switching to differentiation medium (Ham’s F10 with 2mM L-glutamine, 100 μg/mL penicillin/streptomycin, 6% horse serum) for 8 d. Media were renewed every 2 d. One concern in the generation of primary myogenic cultures is obtaining cultures that are free from the most likely contaminating cell type – fibroblasts^36,37^. Our isolation technique has consistently produced cultures in our lab that produce desmin positive multinucleated myotubes (Figs 1,2) that are negative for the fibroblast marker TE7^38^ (Supplementary Fig. 7).

Additionally, commercially available primary human myoblasts (Thermo Fisher cat. No. A12555), isolated from a female aged 21 yr were used for the immunoblotting work presented in Fig. 3 and Supplementary Fig. 4. They were cultured in the same media and conditions as the cultures that we isolated in-house.

### Immunofluorescence Staining

Myotubes were differentiated for 8 d in 24-well culture plates in the presence of rIL-15 (0, 1, 25, 100 ng/mL), rTNFα (0, 1 ng/mL) or both cytokines (rIL-15 25 ng/mL and rTNFα 1 ng/mL). Media were renewed every 2 d. The culture medium was removed and the cells fixed with 2% formaldehyde in PBS for 30 min. Following permeabilization in 100% methanol for 10 min, wells were blocked with 5% goat serum in PBS for 30 min. Primary antibodies were diluted (Desmin, 1:1000, Dako) in 1% BSA/PBS and 150 μL was added per well for 1 hour. Wells were subsequently incubated with 150 μL/well secondary antibody (Goat anti-Mouse IgG (H + L), Alexa Fluor® 488 conjugated, Thermo Fisher) for 1 hour in the dark. Each well was washed with PBS and 150 μL/well DAPI/PBS (1:5000, Cell Signalling Technology) was added for 5 min in the dark. Wells were further washed with PBS, a drop of mountant added to each well (ProLong Diamond Antifade, Thermo Fisher) and a coverslip applied.

### Quantification of Myotube Thickness, Myotube Number and Nuclear Fusion index

24-well plates of IF stained myotubes were imaged on an epifluorescence/brightfield microscope (Leica DMI6000). Triplicate wells were stimulated for each biological replicate and for each treatment condition. Multiple images were taken in each well for the quantification of MTT and NFI. For quantification of MTT, 15 images of each well were obtained using the 63x objective, the first image being obtained at a fixed starting point and subsequent images selected by moving to the next field of view in a predefined pattern. For assessment of NFI, 5 images of each well were obtained in the same fashion, using the 20x objective. Image analysis was carried out by a blinded researcher, using Image J software^39–52^. A myotube was defined as a desmin positive object, containing 3 or more nuclei. The MTT each myotube was calculated by taking the average of 5 measurements obtained along its length. The NFI was defined as the number of nuclei clearly incorporated into myotubes expressed as a proportion of the total visible nuclei in each field of view.

### Real Time Quantitative Polymerase Chain Reaction

RNA was extracted using TRIzol® Reagent (Life Technologies) according to the manufacturer’s instructions. A microvolume spectrophotometer (Nanodrop 2000, Thermo Fisher) was used to quantify each RNA sample and to determine its quality. RNA was reverse transcribed, amplified and quantified in triplicate using Precision Onestep qRT-PCR Mastermix premixed with SYBR greem (Primerdesign) on a Lightcycler 480 instrument (Roche). The primer sequences for each gene are detailed in Supplementary Table 1. Relative RNA expression was determined by the ddCT method, normalized to *ACTB* expression.

### Immunoblotting

For protein extraction, myotubes were lysed in ice-cold RIPA buffer (50 mM Tris HCl, 150 mM NaCl, 1.0% (v/v) NP-40, 0.5% (w/v) Sodium Deoxycholate, 1.0 mM ethylenediaminetetraacetic acid [EDTA], 0.1% (w/v) sodium dodecyl sulfate [SDS], 0.01% (w/v) sodium azide, pH of 7.4), containing a protease and a phosphatase inhibitor cocktail. Total protein lysates of known concentration (bicinchoninic acid assay, Thermo Scientific) were prepared in 4x Laemmli sample loading buffer (0.5 M Tris-HCl pH 6.8, 20% (v/v) Glycerol, 10% (w/v) SDS, 0.1% (w/v) bromophenol blue, 10% (v/v) beta-mercaptoethanol [βME]) and water to generate loading stocks of known concentration. Samples were boiled for 5 min before loading. SDS polyacrylamide gel electrophoresis (SDS-PAGE) gels (12% w/v) were cast in empty gel loading cassettes (Thermo Fisher)

Equal amounts of total protein were loaded onto SDS-PAGE gels. Proteins were separated by electrophoresis (50 mA) in 1x Tris-Glycine SDS-PAGE Buffer (0.025 M Tris base, 0.192 M glylcine, and 0.1% (w/v) SDS). Proteins were then transferred onto polyvinylidene difluoride (PVDF) membrane (Amersham Hybond-P, GE Healthcare) in an electroblotting buffer (0.025 M Tris base, 0.192 M glylcine, 20% (v/v) methanol at pH 8.4), using the ‘Standard SD’ program of the Trans-Blot Turbo semi-dry transfer system (Bio-Rad). Membranes were blocked for 1 h and agitated overnight with primary antibody diluted in tris buffered saline with 0.1% Tween 20 (TBS-T; 50 mM Tris hydrochloride [Tris-HCl], 150 mM sodium chloride [NaCl], 0.1% (v/v) Tween 20 at pH 7.5) at 4 °C (Supplementary Table 2). The membranes were washed for 4 × 5 min in 1xTBS-T and agitated for 1 h at room temperature with HRP-conjugated secondary antibody (Supplementary Table 2). TBS-T washes were repeated and the membranes incubated with ECL Prime Western blotting detection reagents (Amersham Biosciences) according to the manufacturer’s instructions. Blots were visualised on the ChemiDoc MP imaging system (Bio-Rad).

### Enzyme-Linked Immunosorbent Assay

Myotubes were stimulated with 20 ng/mL TNFα. Cell culture supernatants were collected and stored at −80 °C. Supernatant IL-15 concentrations were quantified using Human Quantikine ELISA kits (R&D Systems), according to the manufacturer’s instructions.

### Cell Proliferation Assay

Myoblasts were seeded onto 0.2% gelatin-coated 96-well plates at a density of 2000 cells per well and were stimulated with the indicated recombinant cytokines (IL-15, 25 ng/mL; TNFα, 1 ng/mL in myoblast growth medium). The medium was changed every 48 h and the number of viable cells in culture assessed at days 1, 3, 6 and 9 using the CellTiter 96® Aqueous One Solution MTS Cell Proliferation Assay (Promega) according to the manufacturer’s instructions.

### Non-Radioactive Surface Sensing of Translation (SUnSET) Protein Synthesis Assay

A SUnSET protein synthesis assay was employed^18^. Myotubes were differentiated for 2 d in 24-well culture plates in the presence of rIL-15 (0, 1, 25, 100 ng/mL), rTNFα (0, 1 ng/mL) or both cytokines (rIL-15 25 ng/mL and rTNFα 1 ng/mL). At 2 d, the culture medium was removed and replaced with differentiation medium containing 1 μM puromycin (Sigma Aldrich) for 30 min. Cultures were then prepared for immunoblotting (see above) and the resulting blots were probed with an anti-puromycin primary antibody (Supplementary Table 2). Protein synthesis was estimated by densitometric analysis of each lane, corrected for ponceau stain densitometry values.

### Statistical Analysis

Data analysis was carried out using IBM SPSS Statistics 21. All data are presented as means ± SEM. The normality of data was established by a Shapiro-Wilk test, whereas Levene’s test was used to establish equality of variances. For parametric data involving two treatment conditions unpaired t tests were used. Non-parametric data were analysed by Mann-Whitney U tests. Where data involving more than two treatment conditions were normally distributed, comparison was performed by a one-way or two-way analysis of variance (ANOVA) with post-hoc Bonferroni correction. Where such data were non-parametric, differences between conditions were analysed by Mann –Whitney U test with post-hoc Holm’s sequential Bonferroni correction. A p value of <0.05 was considered statistically significant. Details of the statistical tests used for each data set can be found in the relevant figure legend.

## Electronic supplementary material

Supplementary information
