# Supplementary material for: IL-15 promotes human myogenesis and mitigates the detrimental effects of TNFα on myotube development
Source: Sci Rep. 2017 Oct 11;7:12997. doi: 10.1038/s41598-017-13479-w (PMC5636823; doi:10.1038/s41598-017-13479-w)
Supplement: Supplementary file 1 — Supplementary information [file 41598_2017_13479_MOESM1_ESM.pdf]

Supplementary Information

IL-15 promotes human myogenesis and mitigates the detrimental effects of TNFα on myotube development.

Mary F. O'Leary[1], Graham R. Wallace[1], Andrew J. Bennett[2], Kostas Tsintzas[3*], Simon W. Jones[1*]

[1]Institute of Inflammation and Ageing, University of Birmingham, Birmingham, UK

[2]FRAME Alternatives Laboratory, Faculty of Medicine & Health Sciences, University of Nottingham, Nottingham, UK

[3]MRC-ARK Centre for Musculoskeletal Ageing Research, Faculty of Medicine & Health Sciences, University of Nottingham, Nottingham, UK

Supplementary Figure 1. Recombinant IL-15 stimulation of primary human myoblasts does not alter myogenic regulatory factor expression. A, B, C) Primary human myoblasts from young subjects were stimulated with recombinant IL-15 at the indicated concentrations for 8 d. Media were renewed every 2 d. RNA was extracted by the TRIzol® method and *MYOD/MYOG/MYF5* relative gene expression were quantified by SYBR green RT-PCR. Data expressed as mean ± SEM. 3 technical replicates (each assayed in triplicate) per biological replicate, 3 biological replicates.

Supplementary Figure 2. Recombinant TNFα and IL-15 stimulation of primary human myoblasts does not alter their proliferation or survival. **A)** Primary human myoblasts from young subjects were seeded to 96 well plates and number of viable cells in culture was assayed by colorimetric MTS assay at several time points. Data expressed as mean ± SEM. 4 technical replications per biological replicate, 3

biological replicates. **B-E)** Subconfluent myoblasts from young (white bars) and old (black bars) subjects were switched to differentiation medium containing the indicated recombinant cytokines (IL-15, 25 ng/mL; TNFα, 1 ng/mL) for 8 d. Media were renewed every 2 d. Myotubes were fixed, immunofluorescence stained for desmin and DAPI and imaged on an epifluorescence microscope. Nuclear number data represents the mean ± SEM values from 45 images taken at 20x magnification (from 3 biological replicates). Number of myonuclei per myotube is expressed as mean ± SEM values from 15 images taken at 20x magnification from 3 biological replicates.

Supplementary Figure 3. Representative images of recombinant TNFα and IL-15 stimulation of young and old myogenic cultures. Subconfluent myoblasts were switched to differentiation medium containing the indicated recombinant cytokines (IL-15, 25 ng/mL; TNFα, 1 ng/mL) for 8 d. Media were renewed every 2 d. Myotubes were fixed, immunofluorescence stained for desmin and DAPI and imaged on an epifluorescence microscope.

Supplementary Figure 4. Recombinant IL-15 and TNFα stimulation of differentiating primary human myogenic cultures does not substantially alter protein synthesis. Commercially available subconfluent myoblasts from a female aged 21 yr were switched to differentiation medium containing the indicated recombinant cytokines (IL-15, 25 ng/mL; TNFα, 1 ng/mL) for 2 d. **A)** The expression of total myosin heavy chain was determined by immunoblotting. A full-length blot is presented in Supplementary Figure 5. **B)** At 2 d, cultures were incubated with 1 uM puromycin for 30 min. The incorporation of puromycin into peptides was quantified

by immunoblotting for puromycin. Data are expressed as mean ± SEM, N = 3 independent experiments. A representative immunoblot is also presented.

Supplementary Figure 5. Antibody neutralisation of IL-15rα does not alter the number of myonuclei per myotube. Subconfluent myoblasts were switched to differentiation medium containing the indicated recombinant cytokines and antibodies (IL-15, 25 ng/mL; TNFα, 1 ng/mL; IL-15rα antibody, 1µg/mL; IgG1 antibody, 1µg/mL) for 8 d. Media were renewed every 2 d. Myotubes were fixed, immunofluorescence stained for desmin and DAPI and imaged on an epifluorescence microscope. Number of myonuclei per myotube is expressed as mean ± SEM values from 15 images taken at 20x magnification from 3 biological replicates.

Supplementary Figure 6. Full versions of all cropped immunoblots presented in this paper. Full versions of all myoblast and myotube immunoblots presented in this paper. Stimulation conditions from left to right: unstimulated, IL-15 (25 ng/mL), TNFα (1 ng/mL), IL-15 (25 ng/mL) + TNFα (1 ng/mL).

Supplementary Figure 7. Primary Human Myoblasts do not express the fibroblast marker TE-7. Myoblasts and synovial fibroblasts were fixed, immunofluorescence stained for TE-7 (red) and with DAPI (blue) and imaged on an epifluorescence microscope. A) Synovial fibroblasts, B) Primary human myoblasts.

Supplementary Table 1 - RT-qPCR primer sequences

* Primer sequences not disclosed by Primer Design

Supplementary Table 2 – Immunoblotting antibody details

Supplementary Figure 1

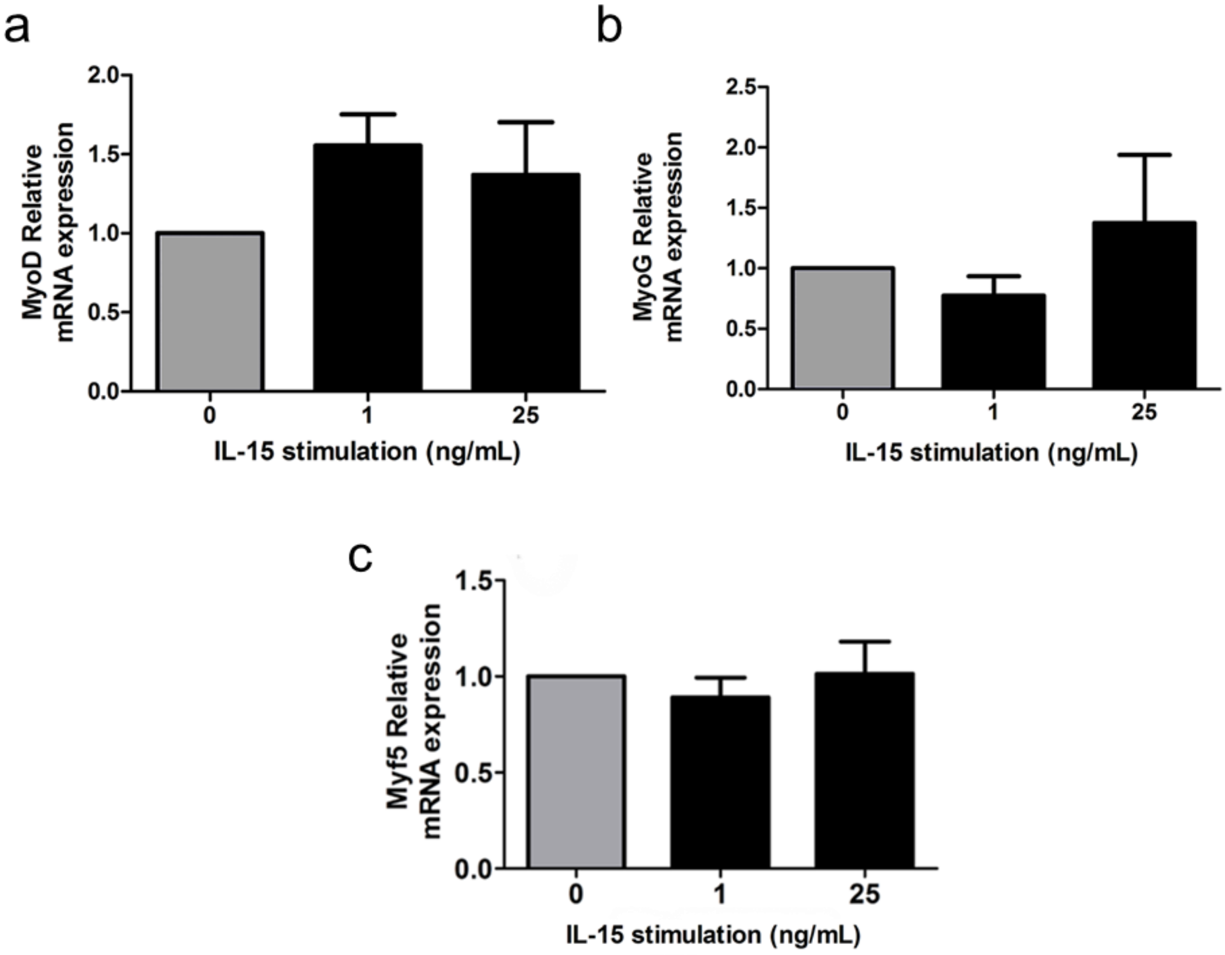

Supplementary Figure 2

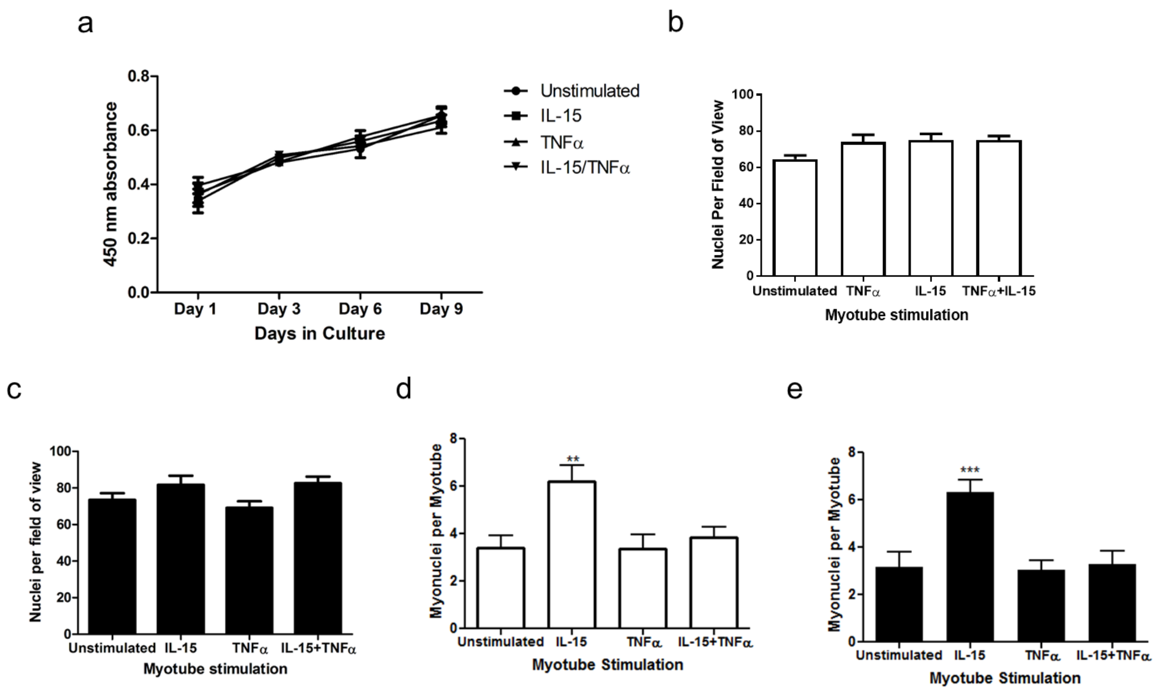

Supplementary Figure 3

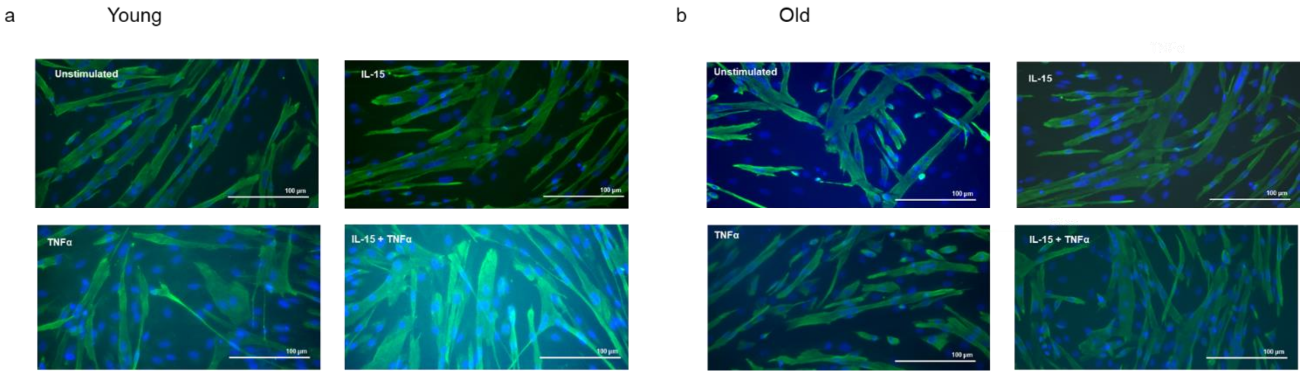

Supplementary Figure 4

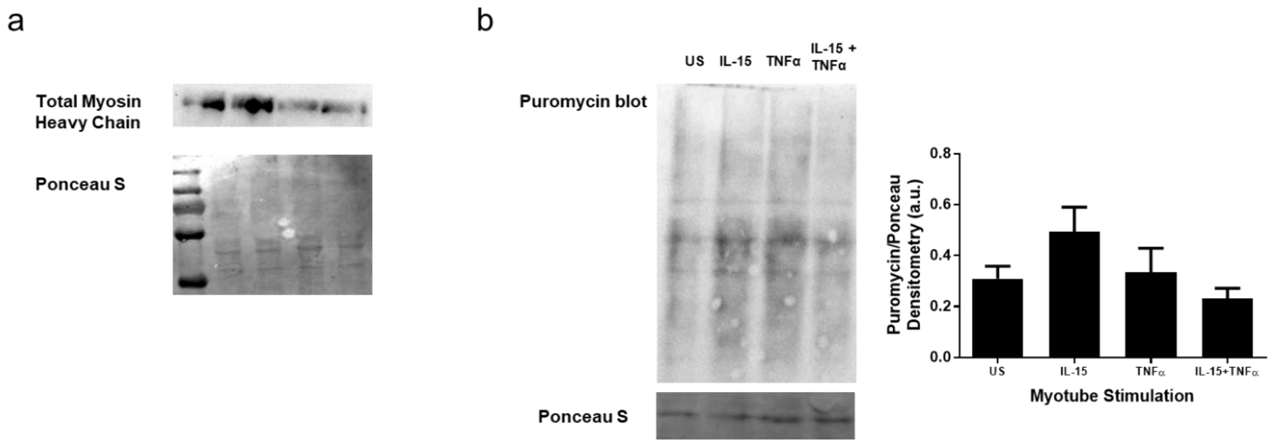

Supplementary Figure 5

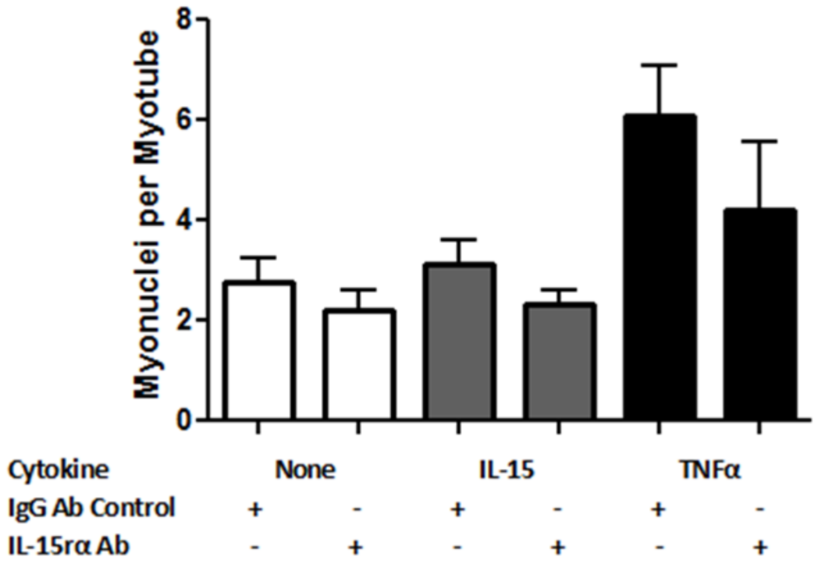

Supplementary Figure 6

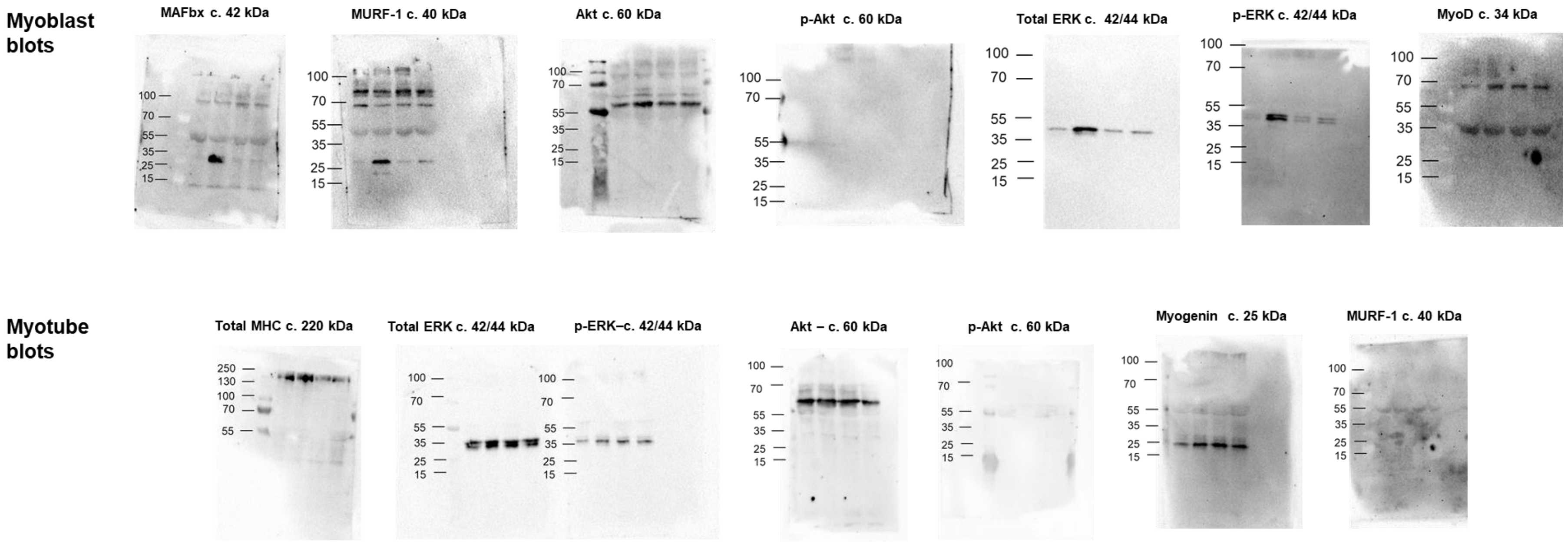

Supplementary Figure 7

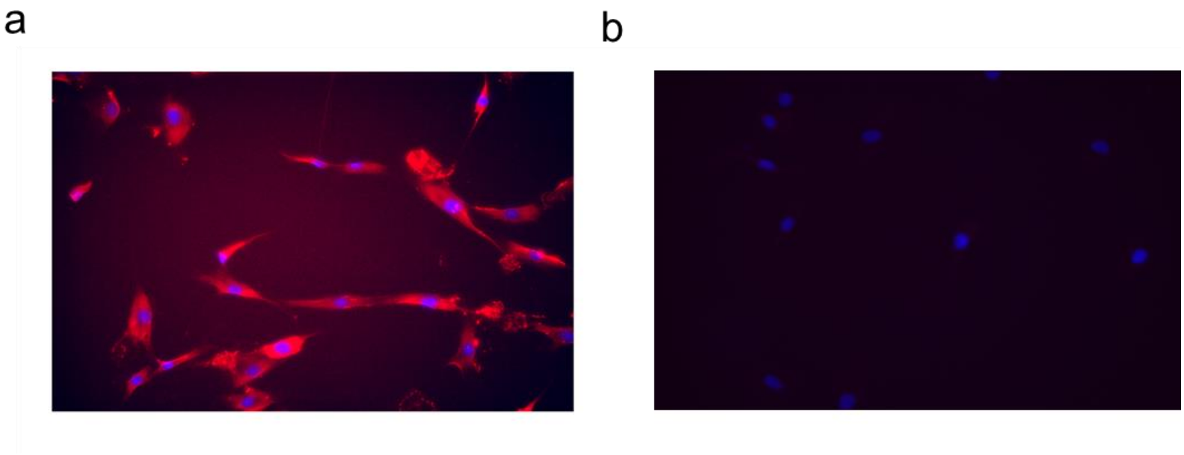

Supplementary Table 1

Gene	Manufacturer	Primer Sequence
ACTB	Primer Design	*
MYOD1	Primer Design	5’-CGCCTGAGCAAAGTAAATGAG-3’ 5’-GCCCTCGATATAGCGGATC-3’
MYOG	Primer Design	5’-GCCCYGATGCTAGGAAGCC-3’ 5’-CTGAATGAGGGCGTCCAGTC-3’
Myomaker	Primer Design	5’-AACTCAAATACAAAATAGGACGCTTT-3’ 5’-CCTTCGCCTTCTCAAAACAAAC-3’
IL15	Primer Design	5’-GGAAACCCCTTGCCATAGC-3’ 5’-GATGGAAATACTTCTCAAATGTGGT-3’
IL15RA	Life Technologies (in-house design)	5'-CGCTGGGCTCAGCATCTC-3' 5'-AGCTGCTCTGCACACATGGA-3'
IL2RB	Life Technologies (in-house design)	5'-GGCTACCTCTTGGGCATCTG-3' 5'-TCGAGTTGTAGAAGCATGTGAACTG-3'

Supplementary Table 2

**Protein**	**Primary Antibody**	**Dilution (diluent)**	**Blocking Agent**
ERK	Cell signaling technology (#9102) rabbit polyclonal	1:1000	(5% BSA in TBS-T)
Phospho-ERK (Thr202/Tyr204)	Cell signaling technology (#9101) rabbit polyclonal	1:1000	(5% BSA in TBS-T)
Akt	Cell signaling technology (#4691) rabbit monoclonal	1:1000	(5% BSA in TBS-T)
Phospho-Akt (Ser 473)	Cell signaling technology (#9271) rabbit polyclonal	1:1000	(5% BSA in TBS-T)
Myogenin	Abcam (ab124800) rabbit monoclonal	1:1000	(5% BSA in TBS-T)
MyoD1	Abcam (ab126726) rabbit monoclonal	1:1000	(5% milk in TBS-T)
MAFbx	Abcam (ab168372) rabbit monoclonal	1:2000	(5% milk in TBS-T)
MURF-1	ECM Biosciences (MP3401) rabbit polyclonal	1:1000	(5% milk in TBS-T)
Total myosin heavy chain	R&D Systems (MAB4470-SP) rabbit monoclonal	1:2000	(5% BSA in TBS-T)
Puromycin	Millipore (MABE343) mouse monoclonal	1:2000	(5% BSA in TBS-T)

Anti-rabbit secondary antibody: 1:15000 Sigma-Aldrich, anti-rabbit IgG HRP linked whole antibody (from donkey) GE NA934V. Anti-mouse

secondary antibody: 1:15000 1:5000 Sigma Aldrich, anti-mouse IgG HRP-linked whole antibody (from sheep) GENA931. BSA = bovine serum albumin.
